# Mechanism-Driven Features Enable Asn Deamidation Reactivity Prediction via Machine Learning Methods

**DOI:** 10.1021/acs.jcim.5c01386

**Published:** 2025-09-19

**Authors:** Maria Laura De Sciscio, Rosa De Troia, Joann Kervadec, Fabio Centola, Simona Saporiti, Muriel Priault, Marco D’Abramo

**Affiliations:** † Department of Chemistry, University of Rome, Sapienza, P.le A. Moro 5, 00185 Rome, Italy; ‡ CNRS, UMR 5095, Institut de Biochimie et de Génétique Cellulaires, Université de Bordeaux, 33077 Bordeaux, France; § Analytical Excellence and Program Management, Merck Serono S.p.A., 00012 Rome, Italy

## Abstract

The spontaneous deamidation of Asparagine (Asn) residues is a common post-translational modification of proteins that can occur on disparate time scales, ranging from hours to thousands of years. This variability in the reaction rate reflects the influence of structural and environmental factors on the multistep mechanism of the deamidation reaction. Understanding the fine connection between reactivity and these modulating factors is essential to advance our knowledge of the deamidation kinetics in proteins and improve the prediction of deamidation-prone residues. In this work, we assessed the step-specific structural-dynamics parameters underlying the chemical basis of the first two reaction stages (the deprotonation and ring-closure steps) and developed novel descriptors derived from molecular dynamics (MD) simulations, which encompass solvation, hydrogen bonds, conformational free energy, and an environment electrostatic effect. These descriptors were evaluated across 63 Asn residues from six distinct proteins and used as input features for three machine learning models, Random Forest, Naive Bayes, and Logistic Regression, to classify Asn residue reactivity. Among these, the Random Forest classifier achieved the best predictive metrics, underscoring the significance of mechanism-tailored features in discriminating Asn reactivity and unveiling the key physicochemical factors that govern deamidation rates in proteins.

## Graphical abstract

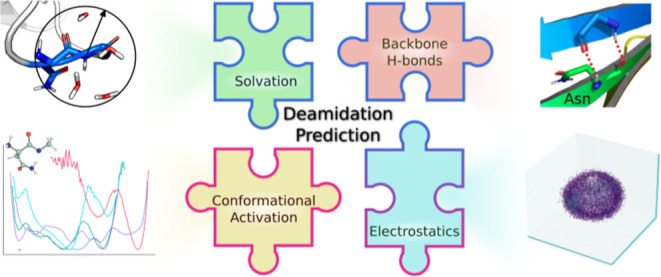

## Introduction

Asparagine (Asn) deamidation is a prevalent degradation pathway of proteins, playing a pivotal role in several physio-pathological processes, including neurodegenerative conditions and cell aging.^1–4^ In the growing market of pharmaceutical products, this spontaneous post-translational modification (PTM) represents a crucial Critical Quality Attribute (CQA) as it can compromise the efficacy and safety of biotherapeutics under development.^5,6^

Protein deamidation under physiological conditions does not require any enzyme. It implies first that both its regulation and kinetics are necessarily different from enzyme-catalyzed reactions; it also implies that under physiological conditions, the activation energy of the reaction is low enough for spontaneous initiation. Also, regardless of the physiological or nonphysiological conditions, this reaction responds to chemistry and physics: the rates of deamidation are profoundly influenced by a variety of environmental factors, comprising pH, temperature, and buffers,^4,7–9^ together with protein primary, secondary, and tertiary structure.^10–15^ Asn deamidation indeed occurs on different time scales and has been proposed as a molecular clock for proteins in vivo.^11,16^ All of these parameters make each Asn in each protein unique in terms of chemistry, and it is therefore imperative to define what discriminates between reactive and nonreactive Asn. External parameters (pH, temperature) have already been investigated in a systematic manner on peptides.^17^ But internal parameters can hardly be investigated by systematic approaches, implying that rationalizing “descriptors” is the key to push beyond our current evaluation of deamidation and assist its prediction.

In neutral-to-basic conditions, deamidation proceeds through a multistep mechanism (see Figure 1A) that involves the initial deprotonation of the backbone NH group on the Asn following (*n* + 1) residue, followed by an intramolecular cyclization to form a metastable tetrahedral intermediate, referred to as Tet^–^. Subsequent expulsion of ammonia results in the formation of a rather stable succinimide intermediate (Suc), the hydrolysis of which yields two reaction products: aspartate (Asp) and isoaspartate (iso-Asp). The formation of the Suc intermediate is the rate-determining step of the overall deamidation reaction, as demonstrated by experimental and computational data.^7,18,19^

**Figure 1:**
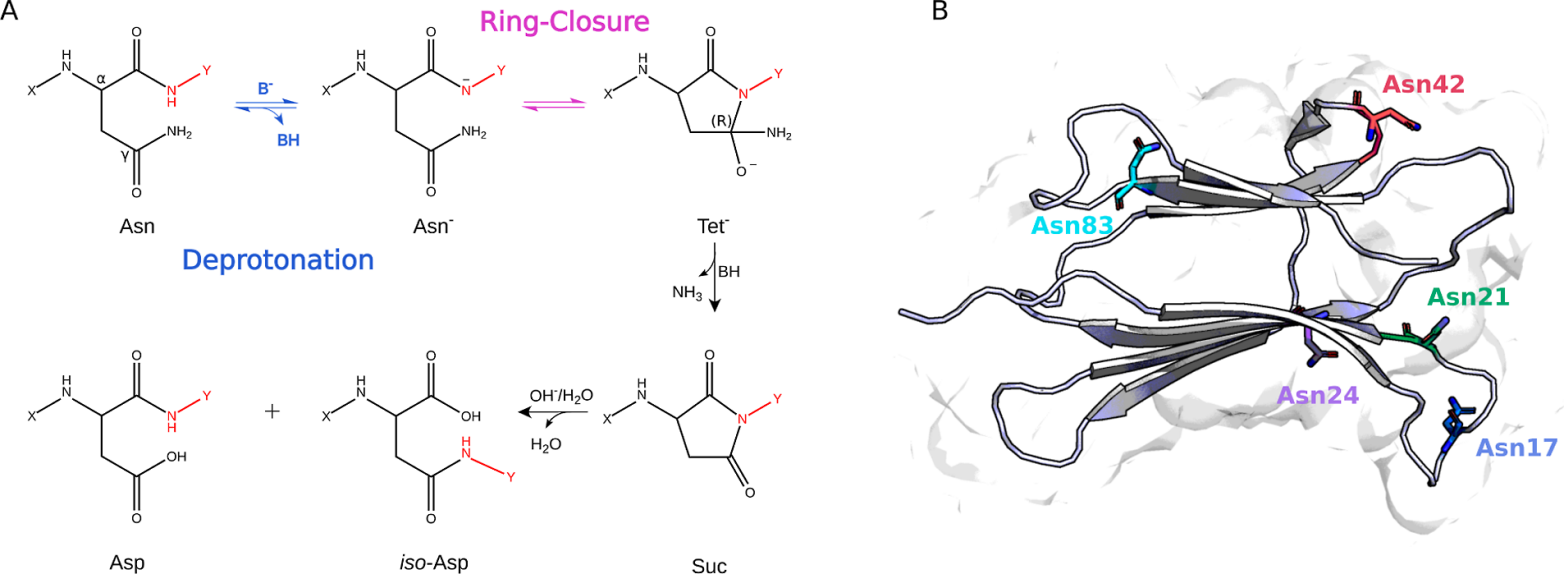
Key reaction steps in dissecting Asn reactivity within B2M as a case study. (A) Deamidation reaction mechanism at nonacidic pH. Atoms in red represent *n* + 1 amino acid, while X and Y are the C-terminal residues and the N-terminal one from the C_α_ atom, respectively. The Asn, Asp, and *iso*-Asp residues are shown in their natural L-configuration. The most relevant steps of the reaction in determining the overall deamidation rates in proteins (deprotonation and ring-closure steps) are highlighted in blue and magenta, respectively. (B) B2M Asn residues shown in sticks and mapped onto the protein structure in cartoon.

Despite the relevance of pinpointing the residue-specific propensity to undergo deamidation in pharmaceuticals,^20^ biology,^21^ and archeology,^22,23^ the multistep mechanism of the reaction has hampered the precise estimation of Asn residues’ propensity to deamidate. Indeed, each step may be affected to varying degrees by environmental factors, complicating the establishment of straightforward correlations between the observed rates and individual molecular properties.

Early predictions relied primarily on the protein sequence,^20^ drawing from experimental observations of extended deamidation in AsnGly sequences and, to a lesser extent, AsnSer sequences in pentapeptides.^11^ A remarkable reactivity of Asn (toward deamidation) within these sequences was frequently detected in proteins as well,^24,25^ which has led to their designation as canonical or deamidating-prone motifs. However, deviations from such canonical motifs have been observed in proteins,^25^ either for missing deamidation in hotspots (i.e., AsnGly, AsnSer) or for observed deamidation with different residues in position *n* + 1. These inconsistencies, along with the limits of sequence-based algorithms in capturing noncanonical reactivity, led to the development of more accurate models including structural features.

The parameters commonly employed in the structure-based predictions, typically combined with machine learning models,^3,26–28^ describe (i) the conformation of the Asn backbone and side chain, (ii) solvent accessibility, (iii) the secondary structure, and (iv) the distance between the NH group of the Asn following residue and the Asn side chain amide carbon. The effect of neighboring residue, specifically the *n* + 1 residue, is often included by using the motif deamidation half-time.^11,22,23,26,29,30^ Furthermore, the addition of consistent mass spectrometry (MS) data resulted in an improvement in the model prediction accuracy.^22,26,30^

However, by neglecting the dynamic nature of proteins, the prediction of reactivity is biased by assuming that the crystal structure is representative of the entire conformational basin of the protein. Despite the growth of computational power that has characterized the last decades, the use of physics-based prediction is still limited. Molecular dynamics (MD) simulations provided useful insights for discriminating Asn residues liability in specific case-study proteins,^31,32^ whereas the use of equilibrium MD-derived structural-dynamics features, to replace the static structure-based predictions, remains underutilized.^33,34^ Recent work demonstrated that the accuracy prediction of the MD conformational ensemble proved to be significantly superior to that of static structures or properties calculated on conformations generated via normal-mode analysis.^35^

A significant concern in building a robust AI-based model is the limited size of publicly available databases on deamidation.^36^ For example, one data set contains the deamidation rates of Asn residues and the amino acid sequences of 131 monoclonal antibodies.^25^ However, as the structures for these antibodies have not been determined experimentally, structural modeling is required, posing particular challenges concerning the complementarity-determining regions (CDRs),^37^ which undergo rearrangements on the millisecond time scale.^38^ Consequently, the accuracy of the MD-based prediction might be affected by the uncertainty of the structural model.

Furthermore, due to the restricted number of Asn residues undergoing deamidation in vitro for each protein, the data sets are usually strongly unbalanced toward nonreactive events. The phenomenon is particularly pronounced in monoclonal antibodies (mAbs),^3,33^ that typically show one or two reactive residues against tens of nonreactive Asn residues, as in the case of Niu and co-workers’ data set, composed of around 300 reactive residues and 2000 inactive residues.^39^

It is worth noting that in the majority of the developed algorithms, experiment-related descriptors were identified as the most significant feature for accurate prediction.^22,26,27^ Beyond predictive metrics, this evidence suggests that a more detailed understanding of the influence of protein structure and dynamics on the reaction rate is lacking. Building on our previous study of the deamidation reaction mechanism in a model system,^18^ this work presents our effort to develop a computational framework for the analysis of deamidation step-specific features in proteins by dissecting the contributions due to the conformational flexibility, *n* + 1 residue acidity, and environmental factors to deamidation rates.

In the hypothesis that the change in the deamidation rates in Asn residues is mainly associated with the first two reaction stages, namely, the deprotonation and ring-closure step (see Figure 1A), we sought to decipher the effect of proteins’ local environment on the reaction kinetics. For this purpose, we have designed a set of structural-dynamics parameters, estimated by means of long-time-scale MD simulations, to capture the molecular basis of a reactive Asn residue. We performed simulations on a representative set of six proteins for which experimental data on site-specific deamidation under physiological conditions are available. Specifically, we selected human β2-microglobulin (B2M), human growth hormone (GH), bovine pancreatic ribonuclease A (RNase), human *Staphylococcus aureus* protein A (SPA), rabbit triosephosphate isomerase (TPI), and trypsin, each featuring at least one Asn residue undergoing deamidation (summarized in Table S1). These proteins were chosen to represent both canonical and noncanonical deamidation motifs, aiming to assess a broad range of reactive-like behavior. For instance, the small soluble B2M protein (11.7 kDa), which constitutes the noncovalent light chain of the major histocompatibility complex, contains five Asn residues, two of which are followed by a Glycine (Asn17 and Asn42). As shown in Figure 1B, these two AsnGly motifs are located in loop regions, thereby creating two potential hotspots. However, Asn42Gly is protected against deamidation, as evidenced by a deamidation half-time (τ_1/2_) of approximately 347 days.^40^ Under physiological conditions, deamidation is observed only for the Asn17Gly fragment.^40,41^ The integration of such noncanonical deamidation motifs, i.e., a deamidation-resistant AsnGly fragment, allowed us to comprehensively explore the physicochemical determinants beyond deamidation susceptibility.

To assess whether the designed parameters could discriminate Asn liability, we trained three Machine Learning algorithms, namely, Random Forest (RF), Gaussian Naive Bayes (NB), and Logistic Regression (LR). The satisfactory prediction metrics obtained demonstrate that an accurate selection of features based on the physical–chemical behavior of the residues could play a pivotal role in the high-throughput screening of biotherapeutics in the early stage of development.

## Methods

### Proteins Selection

The investigation of the physical–chemical properties of reactive and nonreactive Asn residues was conducted on six proteins, resulting in a data set of 63 total Asn residues (see Table S1). The proteins were selected based on multiple criteria: (i) availability of experimental data in literature confirming specific Asn residue deamidation under physiological conditions; (ii) presence of multiple Asn residues per protein; (iii) availability of experimentally determined protein structure with a (iv) predominantly globular folding; and (v) a size suitable for molecular dynamics simulations. Furthermore, to avoid building a data set mainly constituted by deamidating residues within canonical sequences (i.e., AsnGly), we prioritized proteins featuring noncanonical deamidating motifs. Specifically, we included both deamidating Asn residues not followed by a Gly as well as nondeamidating AsnGly sequences. The most critical constraint was the availability of residue-specific experimental annotation of Asn reactivity under physiological conditions, which hampers the expansion of the data set.

### Simulations Details

All the molecular dynamics (MD) simulations were performed using GROMACS^42^ engine and AMBER99sb-ILDN^43^ as the force field. The initial coordinates of each protein were extracted from the available crystallographic structure, while missing residues (if present) were added using AlphaFold2.^44^ Table S1 reports the PDB codes utilized for the MD simulations. Once its coordinates were extracted, the protein was centered in a cubic simulation box that was large enough to avoid interactions within periodic copies. The box was then filled with TIP3P water molecules and an adequate number of ions (Na^+^ and Cl^–^) to reach a concentration equal to 0.15 M while ensuring a neutral solution. The steepest descent algorithm was employed to perform an initial energy minimization. A series of short *NVT* equilibration steps was then performed, and the simulation box volume was tuned to reproduce the water experimental density, as previously described.^45,46^ For each protein, three independent runs, each lasting 300 ns, were then performed in the *NVT* ensemble on the equilibrated structure. The temperature was kept constant at 310 K by the velocity-rescale algorithm.^47^ For all of the simulations, the leapfrog integrator using a time step of 2 fs was employed. Electrostatic interactions were estimated through the Particle Mesh Ewald (PME) method,^48,49^ with a cutoff of 1.1 nm. The same cutoff value was applied for the van der Waals interactions.

### Features Extraction from MD Trajectories

The set of structural-dynamics properties utilized in this work to describe deamidation propensity was computed from MD simulations using either GROMACS or MDTraj library.^50^ After verifying the convergence of the descriptors among the replicates (showing low statistical errors), we used a 750 ns (i.e., the last 250 ns of each run) portion of the trajectory for the analysis of each system.

#### Conformational Analyses

To account for the potential conformational free energy barriers, the parameters usually monitored along the trajectories comprise a set of backbone and side chain dihedral angles of the Asn residue (ψ: N–C_α_–C–N; ϕ: C_*n*–1_–N–C_α_–C; ϕ_*n*+1_: C–N_*n*+1_–C_α,*n*+1_–C_*n*+1_; χ_1_: N–C_α_–C_β_–C_γ_; χ_2_: C_α_–C_β_–C_γ_–N_γ_; δ: N–C_α_–C–O) and the distance N_*n*+1_–C_γ_.

To improve the efficiency in estimating the conformational barrier while ensuring a robust and accurate evaluation of all of the conformational degrees of freedom within the Asn-*n* + 1 residue fragment, we employed a strategy based on the principal component analysis (PCA) of local conformations. Specifically, the local PCA was performed by extracting the coordinates of all the non-hydrogen atoms of the Asn residue analyzed (N, C_α_, C_β_, C_γ_, N_γ_, O_γ_, C, and O) together with N and C_α_ of the *n* + 1 residue colored in red in Figure 1A. The single fragment trajectories were then concatenated, and covariance was built using as reference geometry two AsnNHMe reactive-like conformations, obtained through Quantum Mechanics calculation performed in our previous study.^18^ Specifically, the first geometry represents the reactant state of the ring-closure step (Asn^–^, R) while the second resembles the metastable intermediate Tet^–^ (R*), with the only difference in the distance N_*n*+1_–C_γ_ (see Figures 1A and S3). For each system, PCA was performed on the concatenated trajectories (from the three independent runs), whose convergence was assessed by comparing the sampled conformations within the essential subspace defined by the first two eigenvectors (see Figure S2). Following the projection of the MD samples into the essential space, the free energy profile along the first eigenvector (PC1) was calculated,^51^ referred to as Δ*A*_conf_. For each Asn residue, the free energy values at PC1 ≃ |0.02| and PC1 ≃ |0.05| using R and R* PCA were averaged and used as a feature in the machine learning models, indicated as ${\overset{®}{\Delta A}}_{conf}$ . The free energy profile along PC1 was also calculated on each independent run to further assess the convergence of this descriptor (Figure S4). The selected PC1 value was chosen as representative of a reactive-like conformation. Note that, in all the PCA analyses, the essential subspace was always defined by the first two eigenvectors, spanning consistently on the same range. This evidence is a consequence of the limited number of nonheavy atoms on which the PCA is performed (10 atoms), whose motion is restricted to a small set of degrees of freedom.

#### Asn-*n* + 1 NH Solvation

The solvation level of the backbone NH of each Asn *n* + 1 residue was estimated using the coordination number (CN) equation

1 $$CN=4\pi \int_{r_{0}}^{r_{1}}r^{2}g\left(r\right)\rho \;dr$$

where *g*(*r*) is the radial distribution function of the O atoms of water molecules from the N_*n*+1_ atom. *r* is the distance from N_*n*+1_, *r*_0_ was set as 0 while *r*_1_ as 0.4 nm; ρ is the density of the water molecules calculated as the number of water molecules in the simulation box volume.

#### Hydrogen Bond Analysis

For each Asn residue, the hydrogen bonds established by the CO group of the Asn residue and NH group of the *n* + 1 residue and the neighboring residues were assigned according to the Baker–Hubbard method.^52^

#### Effect of the Environment on the Deprotonation Step

The effect of neighboring atoms on NH acidity was estimated by analyzing the electric field generated by the partial charges of the atoms surrounding the Asn-*n* + 1 backbone segment for each MD frame. Therefore, one electric field vector per MD frame was computed at the center of mass (COM) of the segment composed of C_α_, C, O, N_*n*+1_, and C_α,*n*+1_ atoms. To quantify the spatial distribution of electric field directions throughout the trajectory, we defined a descriptor, ${Vol}_{\mathrm{E⃗}}$ , corresponding to the volume of the space occupied by the electric field vector heads, obtained by summing the volumes of occupied cubes, defined by a three-dimensional grid (50 × 50 × 50). To check the convergence of the electric field spread among replicates, we compared the number of occupied bins in each independent run (Table S2), while the volume was calculated using the entire 750 ns portion.

#### Effect of the Environment on the Ring-Closure Step

Using a similar approach, the effect of the environment on the activation free energy for the ring-closure step was estimated. In this case, we calculated the scalar product between the electric field, as generated by the atoms surrounding the fragment (composed of Asn residue, N_*n*+1_, and C_α,*n*+1_) and the dipole moment of the gas-phase AsnNHMe reactant state (R) as well as the dipole moment of the gas-phase AsnNHMe transition state (TS). The electronic properties of such states were retrieved from our previous work on deamidation modeling.^18^ A decrease in the activation energy occurs when the environment exerts a larger stabilization on the TS compared with the reactant ((*E*·μ)_TS_ – (*E*·μ)_R_ > 0). The environment effect on reactant and TS was estimated for each frame of the simulation, thereby allowing the estimation of a percentage of frames where the ring-closure step is favored. This analysis was restricted to the MD frames where the sampled conformation resembles the gas-phase reactant state (R), RMSD < 0.15 nm. The resulting values were further normalized on the average conformational free energy (which is a more accurate evaluation of reactive-like conformation rather than RMSD value)

2 $$RCS=\Delta \left(E\cdot \mu \right)\times \left(1-\frac{{\overset{®}{\Delta A}}_{conf}}{\Delta A_{conf}^{\max}}\right)$$

where Δ*A*_conf_^max^ is the maximum conformational free energy of the entire data set; Δ­(*E*·μ) represents the difference between the stabilization of the TS and R species due to the electric field generated by the environment, i.e., Δ­(*E*·μ) = (*E*·μ)_TS_ – (*E*·μ)_R_. We refer to this final descriptor as ring closure stabilization (RCS).

### Data Set Construction and ML Algorithms

The 6 features described above were calculated for each of the 63 Asn residues. In building the data set, Asn residues having a Pro as *n* + 1 residue were excluded because of the lack of the backbone amide. The first Asn residue of *Staphylococcus aureus* protein A (SPA) was also excluded from the data set due to the absence of experimental data. For all of the other residues, no special actions were necessary. The experimental observations were expressed in binary mode (Yes or Not) according to the data available in literature on Asn deamidation in physiological conditions (see Table S1).

For the Asn-*n* + 1 fragment for which no reactive-like conformations were sampled along the MD simulations, we assigned to Δ*A*_conf_ a value of 1 order of magnitude greater than the maximum conformational free energy sampled along the entire data set.

For all the machine learning (ML) methods employed (Naive Bayes (NB), Logistic Regression (LR), and Random Forest (RF)), a fine-tuning of the hyperparameters with 8-fold cross-validation was performed before the training using GridSearch implemented in Scikit-learn (version 1.5.2). The area under the receiver operating characteristic curve (ROC-AUC) was selected as the scoring parameter. For the NB model, the smoothing parameter optimized value was searched from 50 initial values ranging from 10^–9^ to 10. In the LR statistic model, the liblinear solver was employed, and the maximum number of iterations was set to 100 while the (inverse) strength parameter *C* was searched in the range 0.01–10. RF has a higher number of hyperparameters to optimize. Specifically, we fine-tuned the number of trees (range 10–300), the depth of each tree (range 5–30, plus none), the minimum samples required for an internal node division (range 2–10), and the minimum samples required for an external node division (range 1–2). Given the reduced size of the data set and the high number of features, only the square root of the total features and the log_2_ of the total features were tested.

The evaluation of the models was performed by calculating the classes’ nonweighted (due to the imbalance of the data set) average metrics (precision, recall, and *F*1-score). Accuracy, AUC, and the Matthews correlation coefficient (MCC) were also employed in the evaluations. MCC proved significantly useful in imbalanced data sets and for evaluating the overall prediction capability of a model.^53^ A deamidation score was defined as the predicted probability of a residue being reactive derived from model outputs using NB, RF, and LR classifiers. Feature importance was estimated via the leave-one-feature-out (LOFO) method. Briefly, for each of the best models obtained by GridSearch as described above, one feature was iteratively removed from both the (SMOTE-enhanced) training and the test set. The difference between the accuracy achieved by the best model trained with all features and the accuracy attained by the same model but with one feature removed (from the training and testing sets) was evaluated as an indicator of feature relevance. The same methodology was repeated three times, using a different train-test split of the data set, ensuring a preserved ratio of negative and positive events in each split (stratified sampling), as obtained by changing the random seed number.

## Results and Discussion

The deamidation of Asn residues at nonacidic pH necessitates the formation of a succinimide intermediate, which is recognized as the rate-determining step of the reaction. To gain insight into the molecular characteristics that may influence the overall rate of the reaction, we have undertaken a detailed analysis of potential features to describe the first two steps of the deamidation reaction, which we consider essential in discriminating reactive and nonreactive residues.

All of the descriptors were calculated for six proteins, leading to a final database of 63 residues (see Table S1) with 6 observables obtained by all-atom MD simulations. To improve clarity, the selected step-specific features are shown in the next sections using β2-microglobulin (B2M) as a test case.

Given this work’s aim of comprehending the molecular hallmarks of the different deamidation rates in proteins as well as to improve the reactivity prediction by training models based on reaction-tailored features, we applied machine learning models to the generated data set. The satisfactory ability of Naive Bayesian (NB), Random Forest (RF), and Logistic Regression (LR) classifiers to identify the Asn reactivity exclusively based on the designed features was instrumental in highlighting the crucial role of the deprotonation and ring-closure steps on the overall deamidation rates in proteins. Furthermore, it also demonstrates how features designed on the chemical nature of the reactions enable precise reactivity predictions, even without additional knowledge-based parameters (for instance, *n* + 1 residue).

### Backbone N–H Acidity

In neutral to basic conditions, the first step of the deamidation reaction is the deprotonation of the nitrogen atom of the *n* + 1 residue (N_*n*+1_). Li and co-workers^54^ demonstrated, indeed, the essential role of the deprotonation step by substituting the hydrogen atom bound to the N_*n*+1_ atom into a methyl group, leading to complete inhibition of the deamidation reaction. Therefore, the acidity of the nitrogen atom traces the first boundary in discriminating Asn residues’ reactivity.

Although the acidity of the nitrogen atom in the backbone of proteins is still a matter of debate,^55–58^ evidence suggests that the local environment affects the acidity, resulting in significant p*K*_a_ variations between different residues within the same protein.^59^ Furthermore, it was suggested that the acidity of N–H is affected by the backbone conformations,^60^ with a maximum acidity reached for values of ϕ and ψ equal to −180 and 0.^56^ These dihedral angle values were recently proposed by Creutznacher and co-workers^32^ as a deamidation-enhancing conformation.

The neighboring residues and solvation electrostatically affect the backbone acidity. Moreover, the hydrogen bond, including either the oxygen or nitrogen atom, could affect the p*K*_a_ by inductive and steric effects.^61^

In light of these considerations, the estimation of Asn reactivity could be improved by the inclusion of backbone acidity. We have monitored multiple molecular features linked to N–H acidity, aiming to analyze the different contributions to N–H acidity and their effect on deamidation prediction. As the amide proton should be abstracted by a base, for instance, the hydroxyl ion, the first structural aspect to consider is the solvent accessibility of the amide. Using the all-atom simulations in explicit solvent, an accurate estimation of nitrogen solvation has been performed by calculating the radial distribution function (*g*(*r*)) of the water molecules around the N_*n*+1_ atom, reported in Figure 2A. From that, the Coordination Number (CN) for each nitrogen atom was obtained by integrating the number of water molecules within a sphere of 0.4 nm around the N_*n*+1_ atom, according to eq 1 (see Figure 2B). In the B2M case, the Asn-*n* + 1 residues displaying a higher CN value are Asn17Gly, Asn42Gly, and Asn83His (Figure 2C), which are all located in unstructured regions, i.e., in a loop on the protein surface (see Figure 1B). On the contrary, the remaining Asn residues (Asn21Phe and Asn24Cys) are situated close or in the center of the beta-sheets of B2M, constituting the core of the protein (see Figure 1B). Consequently, the deprotonation of these residues should reasonably occur to a lesser extent, thereby slowing or inhibiting the deamidation reaction under physiological conditions.

**Figure 2:**
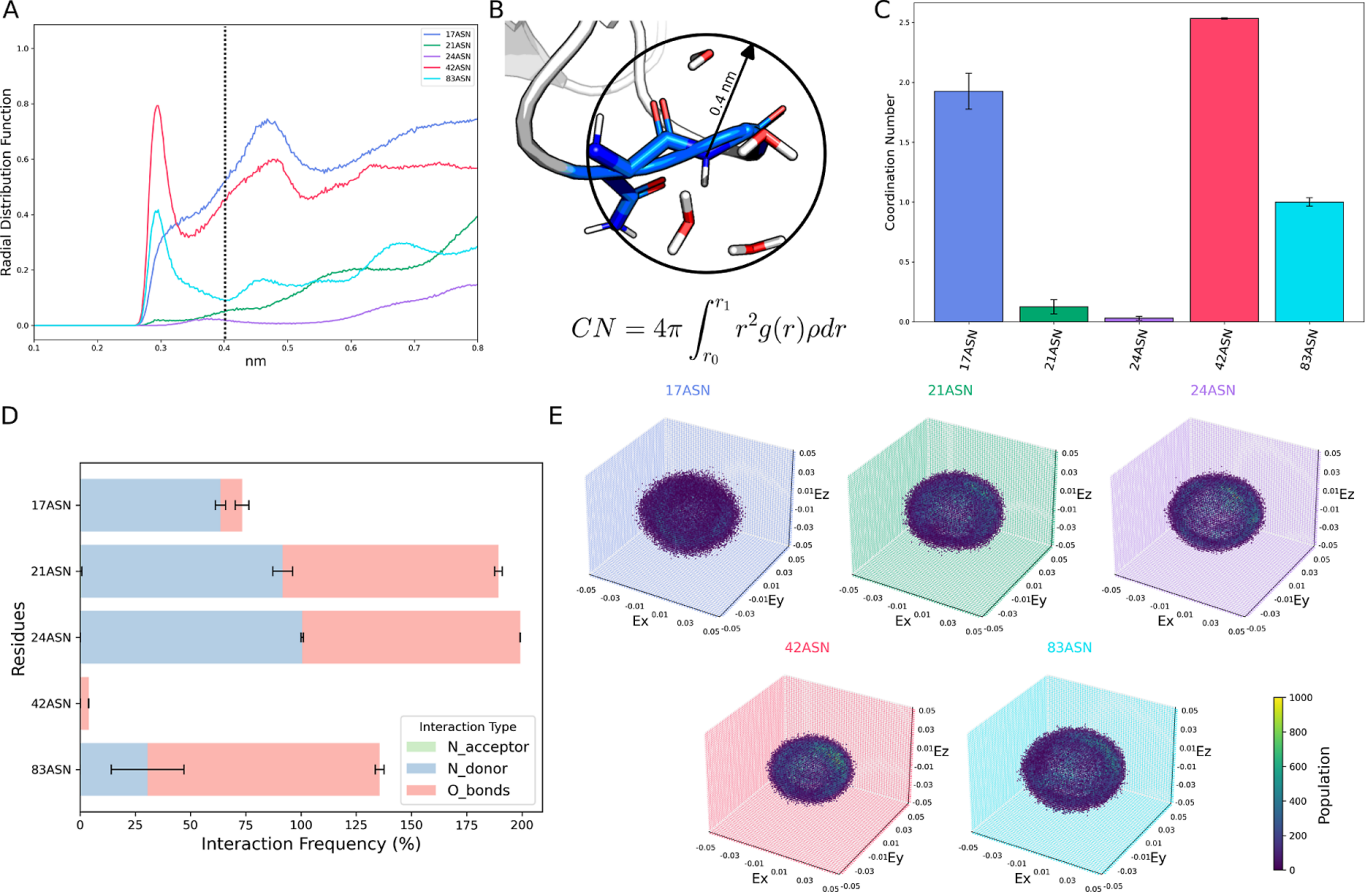
N_*n*+1_–H acidity estimation via MD-derived features. (A) Radial distribution function (*g*(*r*)) of each nitrogen atom potentially involved in the deamidation reaction, i.e., the one following an Asn residue, zoomed in the region 0.1–0.8 nm. (B). Quantification of nitrogen solvation by integrating the radial distribution function between 0 nm (*r*_0_) and 0.4 nm (dashed line in panel (A), *r*_1_). Top row, a representative frame of the solvation of Gly18-N atom within the applied cutoff. In the bottom row, the coordination number (CN) equation utilized to quantify solvation, leading to residue-specific N_*n*+1_ water coordinating number, shown as bar plot in panel (C). (D) Frequency of hydrogen bonds established by Asn-*n* + 1 amide group. Each H-bond type can range between 0 and 100, indicating a complete absence of interaction or an interaction conserved across the entire trajectory, respectively. H-bonds established by the oxygen atom are in red; the ones involving the N atom are in blue (donor) and in green (acceptor). Error bars in panels (C,D) depict statistical error among replicates, represented as half the standard deviation for clarity. (E) Electrostatic effect of neighboring residues, obtained by analyzing the electric field exerted by the residue and the solvent around the Asn-*n* + 1 backbone segment (atoms: C_α_, C, O, N_*n*+1_, C_α,*n*+1_) (see Methods for further details).

The hydrogen bonds established by the polar atoms of the amide group were suggested to influence the acidity of N–H.^61^ However, the interactions established by oxygen or nitrogen may exert opposite effects on acidity. For instance, the hydrogen bonds involving the amide atom should increase the nucleophilicity of the nitrogen. Conversely, if nitrogen acts as a hydrogen donor, the protonated state is stabilized, potentially leading to a decrease in acidity. On the other hand, when the nitrogen atom accepts a hydrogen atom via a hydrogen bond, the anionic state may be stabilized, thereby enhancing acidity. To account for the potential effects of hydrogen bonds, we independently calculated the hydrogen bonds established by either the oxygen or nitrogen atom. The latter were further divided into two classes based on their role in the interactions, that is, whether they acted as a donor or acceptor in the analyzed hydrogen bond. In Figure 2D, the hydrogen bond of B2M Asn-*n* + 1 amide is reported as a percentage of frames showing the specific interaction normalized on the total frames. It should be noted that the percentage has been normalized for every interaction, such that 100% of each interaction corresponds to an interaction retained throughout the whole simulation. Furthermore, the hydrogen-bond analyses also directly include the secondary structure of the protein in which the Asn-*n* + 1 segment is situated. In fact, the two residues in the protein’s core feature stable hydrogen bonds with both the oxygen atom and the nitrogen, indicating the presence of a structured region where the hydrogen bonds are required to form the secondary structure. The deprotonation of such an amide should be, therefore, disfavored.

Finally, the electrostatic effect of the local protein environment on the N_(*n*+1)_–H bond was evaluated by analyzing the electric field generated by the protein residues and solvent. Given the absence of a direct relationship between the acidity and the electric field and the inability to generate it (no experimental data on amide p*K*_a_ in the analyzed proteins is available), we assumed that a larger spread in the sampled electric field might be associated with a higher probability of sampling acidity-enhancing configurations of the environment. Therefore, for each Asn residue, we estimated the volume of the three-dimensional space occupied by the electric field vectors sampled at each MD frame (see Methods for further details), illustrated in Figure 2E. Such volume values, reported in Table S2 for B2M, reflect the environment fluctuations around the segment, and indeed, the highest value was obtained for the flexible, deamidation-prone Asn17Gly segment. Those values were then utilized as a description $\left({Vol}_{\mathrm{E⃗}}\right)$ of the environment effect on the N–H acidity.

### Conformational Activation of the Asn-*n* + 1 Fragment

Once the backbone NH group of the *n* + 1 residue loses the proton, the nitrogen anion attacks the carbonyl carbon of the Asn side chain amide, leading to the tetrahedral intermediate Tet^–^ (see Figure 1A). This ring closure step depends on the local conformational flexibility. Indeed, during the reaction, both the Asn side chain and the Asn-*n* + 1 backbone segment should take a rather specific conformation. Different approaches were used to take into account the conformational aspect in deamidation reactivity.^28,33–35,62^ The most common features implemented in the reactivity-like analyses comprise the distance N–C_γ_ and several dihedral angles, among which ϕ, ψ, χ_1_, and χ_2_ of Asn residue emerged as relevant in previous Machine Learning works.^26,27^

Along the B2M MD simulation, we monitored a total of six dihedral angles and the distance N_*n*+1_–C_γ_, which are illustrated in Figure S1. The two B2M hotspots, Asn17Gly and Asn42Gly, represent an interesting case study to dissect the conformational flexibility role in deamidation. Despite being located in an unstructured region (see Figure 1B), Asn42 conformational flexibility is indeed significantly limited, as demonstrated by the kernel density estimate (KDE) unimodal distribution of most of the geometrical features evaluated, reported in Figure S1. On the other hand, Asn17 exhibits a rather free rotation around multiple bonds, thereby demonstrating a high conformational flexibility.

However, the interpretation of such a large number of conformational variables is not straightforward. Therefore, to reduce the dimensionality of the conformational degrees of freedom, we employed a strategy based on a local Principal Component Analysis (PCA), utilizing the sampled conformations of Asn residues within each protein. The PCA of the non-hydrogen atomic coordinates was constructed using the quantum mechanical (QM)-derived reactive-like conformation of AsnNHMe (Asn^–^ in Figure 1A, R in Figure S3), as obtained in our previous work.^18^ As illustrated in Figures 3A and S2, this approach reduced the computational effort required to identify the conformational barriers from several parameters while simultaneously evaluating all of the correlated degrees of freedom for all of the residues of interest. Furthermore, by constructing a free energy landscape along the first principal component (PC1),^51^ illustrated in Figure 3A, the deviation of the sampled local AsnGly conformations from the reactive-like reference structure could be quantified. Moreover, according to the mechanism described above, the ring-closure reaction step requires rotation of the backbone to form the metastable tetrahedral intermediate (Tet^–^). For this reason, the objective is to evaluate whether a given Asn residue could adopt a backbone conformation conducive to Tet^–^ formation. To capture this conformational rearrangement, we performed an additional PCA using a QM reference structure (R* in Figure S3) in which backbone rotation had already occurred. In both instances, the free energy at a specific value along the projection on PC1, which represents a reactive-like conformation (see Methods), was selected as a feature to describe the conformational distance from a reactive-like state. The resulting free energy profiles along PC1 for all of the Asn residues in B2M are reported in Figures S3 and S4.

**Figure 3:**
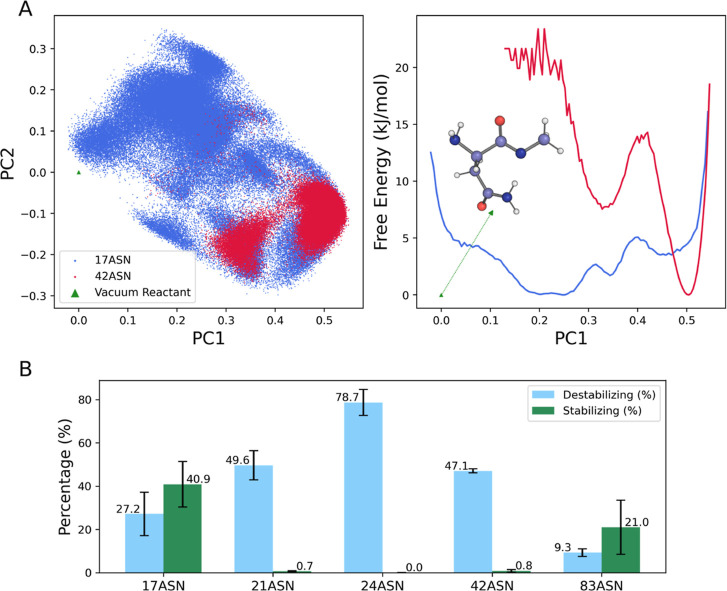
Conformational dynamics of Asn-*n* + 1 in B2M. (A) Local AsnGly conformations projected into the essential subspace defined by the first two PCs, representing 77% of the total variance (left), built on AsnGly reactive conformation as derived by QM calculations, illustrated as ball and sticks. On the right, the free energy variation along the first PC quantifies the conformational reactivity of AsnGly hotspots. (B) Effect of the environment surroundings Asn-*n* + 1 segment on the activation free energy for the cyclization step. Frames where the TS experiences a (de)­stabilization stronger than the reactant state of at least 6 kcal/mol are labeled as (de)­stabilizing. Error bars illustrate the statistical error among replicates, represented as half the standard deviation for clarity.

Notably, Asn17 was the sole residue for which sampled conformations exhibited similarity to both of the QM-derived reference structures.

Even though conformational flexibility is a key prerequisite for the ring-closure step, the effect of the local protein environment could also affect the deamidation rates.^10^

Indeed, the electrophilicity of the side chain amide carbon atom together with the nucleophilicity of N_*n*+1_ might be affected by potential interactions with the surrounding environment, including the neighboring residues and the solvent. The point charges of such atoms generated an electric field that might be oriented as the Asn^–^ dipole moment, leading to a stabilizing effect.

By calculating the difference in the electric field generated by the point charges of the surroundings on the dipole moment of both the reactant state and the ring-closure gas-phase TS, an estimation of the electrostatic effect of the protein–solvent environment on the activation free energy of the ring-closure step was estimated (see Methods). To quantify such a stabilizing effect among the reactive-like conformations, the percentage of frames in which the TS stabilization was higher than the reactant (qualitatively indicating a potential decrease in the activation free energy) was used as a descriptor of the effect of the protein environment on the ring-closure reaction step. In the B2M Asn-*n* + 1 fragments, the electric field generated by the environment exerted a stabilizing effect only for Asn17Gly and Asn83His, as shown in Figure 3B.

### Asn Deamidation Reactivity Prediction

To assess the ability of the developed features to predict residue susceptibility against deamidation, we built a database comprising 63 Asn residues from 6 proteins. Among these residues, summarized in Table S1, 13 of them showed experimental deamidation under physiological conditions. For each Asn residue, six structural-dynamics parameters (Table 1) were assessed concurrently to address reactivity toward deamidation. Indeed, when considered alone, these parameters were unable to differentiate the reactivity of Asn residues, as demonstrated by the overlapping distribution of features among reactive and nonreactive samples (see Figure S5).

**Table 1:** Features Developed for Asn Deamidation and Employed in ML Models^a^

feature	step	group	expected role
CN	deprotonation	solvation	high CN promotes reactivity
${Vol}_{\mathrm{E⃗}}$	deprotonation	electrostatics	large value increases reactivity
% N H-bonds	deprotonation	H-bonds	high % reduces reactivity
% N–H H-bonds	deprotonation	H-bonds	high % reduces reactivity
${\overset{®}{\Delta A}}_{conf}$	ring-closure	conformation	low values increase reactivity
RCS	ring-closure	electrostatics	strong stabilization favors reactivity

a CN = coordination number, RCS = ring closure stabilization; see Methods for further details.

Nonetheless, as illustrated in Figure S6, deamidating Asn residues exhibit—from a chemical point of view—expected values of the features, which are intuitive given the mechanism-based parameters (described in Figure S6 and summarized in Table 1). It is noteworthy that each Asn residue is characterized by a multifactorial mechanism with a different reactive-like pattern, which may, in principle, reflect the diverse deamidation rates.

Even though the number of Asn residues is somewhat limited for a robust machine learning (ML) model, we applied different ML methods, namely, Naive Bayesian (NB), Logistic Regression (LR), and Random Forest (RF), to test the efficiency of the selected features (listed in Table 1) in capturing this multifactorial (non)­reactivity of residues among the selected proteins.

Given the unbalanced data set available (13 reactive vs 50 nonreactive Asn), which is typical in the prediction of deamidation reactivity,^27,39^ we trained the models employing the Synthetic Minority Oversampling Technique (SMOTE).^63^ This oversampling method generates synthetic entries according to the k-nearest neighbors of the class of interest, rather than generating copies of existing data, thus reducing oversampling issues.^63,64^ Consequently, an enhanced capacity to accurately predict reactive residues was attained. The data set was randomly divided into train and test sets with a ratio of 70 and 30%, respectively. Three random divisions of the data set were performed to further validate the prediction metrics. The best results obtained within a testing set are presented in this section. The prediction metrics of such a training set are summarized in Table 2. In addition, the receiving operating curve (ROC) and the corresponding area under the curve (AUC), along with the confusion matrix, are shown in Figure 4B. The results from the additional testing sets, as well as the average metrics, are reported in Table S3.

**Table 2:** Statistic for RF, LG, and NB Predictions on the Test Set^a^

model	precision	recall	accuracy	*F*1-score	AUC	MCC
RF	0.97	0.88	0.95	0.91	0.87	0.84
NB	0.67	0.74	0.74	0.68	0.85	0.41
LR	0.86	0.84	0.84	0.78	0.83	0.57

a Precision, *F*1-score, and recall are obtained from nonweighted averages on both reactive and nonreactive class predictions. MCC = Matthew’s correlation coefficient and AUC = area under the receiving operating curve (ROC). The corresponding curves are reported in Figure 4.

**Figure 4:**
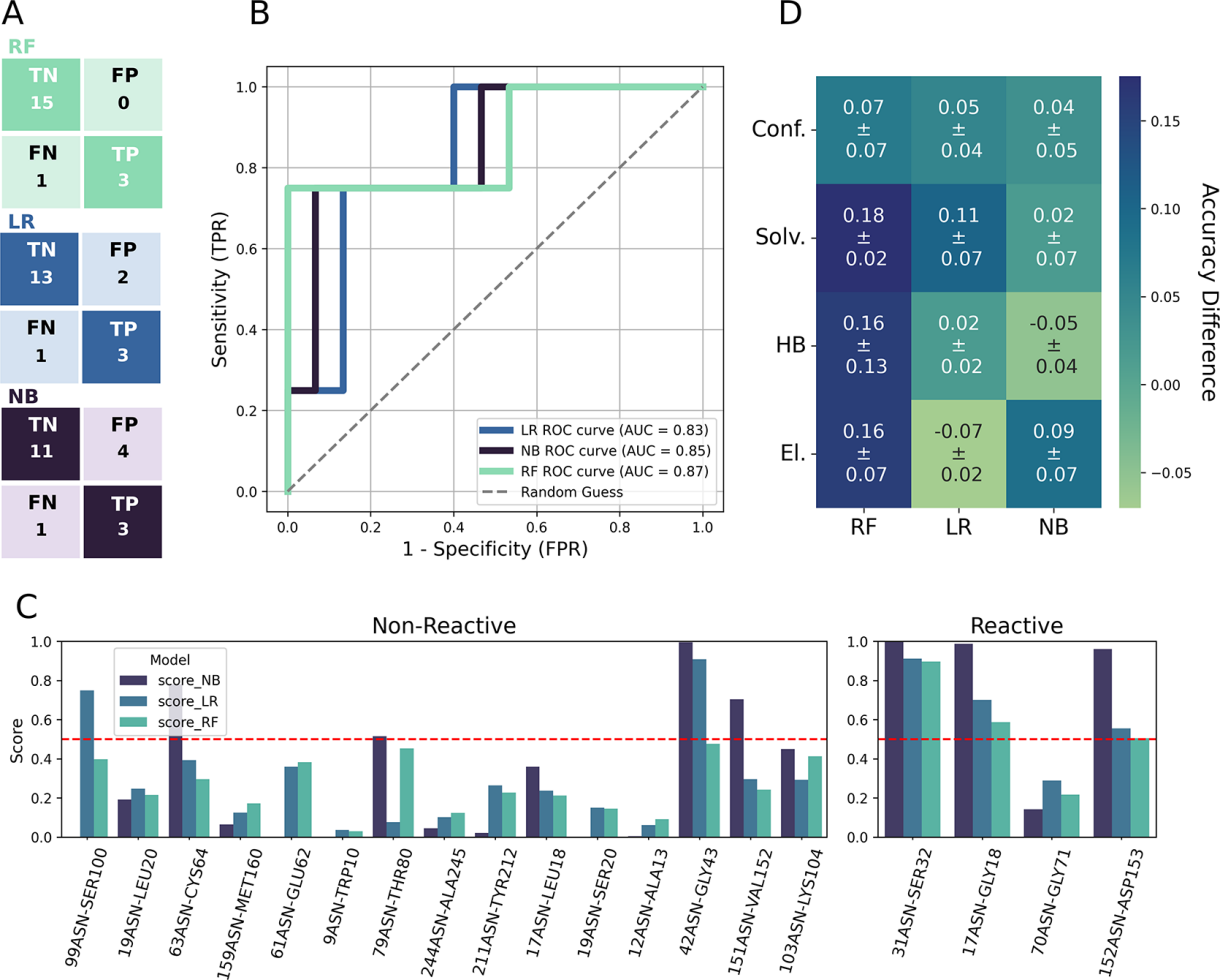
Test set prediction results from NB, LR, and RF models. Confusion matrix and ROC-AUC curves (A,B). (C) Bar plot of the obtained deamidation score for nonreactive (left) and reactive (right) Asn residues within the test set. The red dashed line indicates the cutoff (0.5) utilized for discriminating reactive residues from nonreactive ones. (D) Leave-One-feature-out drop in accuracy for the three ML classifiers utilizing clustered features, grouped by their physical–chemical properties (see Table 1), averaged across the three data set splits. Conf: Conformational, Solv: Solvation, HB: Hydrogen Bonds, El.: Electrostatics, TN: True Negative, TP: True Positive, FP: False Positive, FN: False Negative, TPR: True Positive Rate, FPR: False Positive Rate.

Despite the restricted data set available and the absence of sequence-based descriptors, the attained prediction metrics are rather satisfactory. Among the models, RF consistently outperformed both NB and LG (Tables 2 and S3). Nonetheless, further analyses were performed on all the models to enhance comprehension of the significance of the features. To compare the performance of the three models in predicting site-specific Asn residue susceptibility, we calculated a deamidation score (see Methods), representing the model-assigned probability that an Asn residue is reactive (1) or nonreactive (0). A threshold of 0.5 (indicated by the red dashed line in Figure 4C) was applied to discriminate the residue reactivity. Deamidation scores for the test set residues are shown in Figure 4C and are listed in Table S4.

The RF model correctly identified the reactivity of almost all of the residues, with only one false negative (Asn70 of TPI). Interestingly, it accurately predicted the reactivity of noncanonical hotspots, such as the deamidation-prone Asn152Asp of GH as well as the deamidation-resistant Asn42Gly of B2M. In contrast, NB and LR models both predicted three out of four reactive residues but misclassified Asn42Gly of B2M by assigning to it a high score (representing a deamidation-prone residue). To dissect the contribution of each mechanism-related descriptor, we employed the leave-one-feature-out (LOFO) strategy, which revealed differences in the features’ importance across models (see Figure S7). The RF model’s superior performance is attributable to its capacity to incorporate all of the features implicated in the decision process. Indeed, the removal of any of the designed features resulted in a consistent reduction of the prediction accuracy, corroborating the hypothesis of the coexistence of multiple activating parameters to discriminate Asn reactivity. The solvation of the NH group and the effect of the environment on the ring-closure step, respectively, described by the CN and RCS parameters, were associated with the greatest drop in RF accuracy, underscoring the significance of solvation and conformational-environment factors in predicting Asn reactivity.

In contrast, the majority of the features removed from the NB and LR models resulted in a negligible reduction or even slight increases in accuracy, revealing the limited capacity of these classifiers to incorporate multiple interdependent features concurrently.

When the LOFO strategy is applied to groups of features (see Table 1), the difference between the three classifiers becomes more pronounced. As shown in Figure 4D, the RF model predictions rely on all of the chemically relevant groups, highlighting the comparable weight of each descriptor. Interestingly, the removal of ${\overset{®}{\Delta A}}_{conf}$ provided a limited effect across all the classifiers, despite its central role in describing the ring-closure step (as shown by rather favorable values among deamidating residues; see Figures S5 and S6). This outcome suggests that conformational accessibility is partially captured by other descriptors. Indeed, the Pearson correlation matrix in Figure S8 revealed a moderate (negative) correlation of this local feature with more global parameters, such as the NH solvation (CN) and the effect of the environment ( ${Vol}_{\mathrm{E⃗}}$ and RCS). These correlations reflect the chemical nature of the descriptors: ${\overset{®}{\Delta A}}_{conf}$ and ${Vol}_{\mathrm{E⃗}}$ both inherently include the flexibility and fluctuations; a higher solvation indicate an exposed residue, often more flexible than the core residues, whereas RCS combines conformational activation with environmental electrostatic effects (see eq 2). This evidence suggests that the LOFO results on ${\overset{®}{\Delta A}}_{conf}$ are potentially influenced by the features correlation and that Asn conformational activation may be crucial in describing reactivity.

Altogether, these findings suggest that deamidation reactivity arises from an interplay of different structural-dynamics parameters that should be assessed simultaneously.

## Conclusions

The rates of Asn deamidation in proteins are determined by a complex interplay of structural-dynamics parameters. Despite the development of various machine learning (ML) models in recent years,^3,22,26,27,29,30,35,36^ the predictions largely depended on knowledge-based data, such as the Asn-*n* + 1 segment half-life time in pentapeptides^11^ and mass spectrometry data. In this study, we developed new structural dynamics features tailored for machine learning (ML) applications based on the chemical properties necessary to initiate the reaction. These mechanism-guided descriptors enabled us to hypothesize the parameters potentially affecting Asn deamidation in proteins. These encompass a range of parameters, including conformational parameters such as the conformational free energy, as obtained by PCA of the local conformations of Asn residues sampled during MD simulations. The acidity of the backbone amide NH was analyzed by including the solvation effect, interactions (hydrogen bonds), and environment effect on the stability of the NH bond. Additionally, the electrostatic effect of the neighboring residues on the ring-closure step was investigated. In the case of Asn residues within B2M, Asn17, the most susceptible residue to deamidation, displayed consistent reactive-like behavior across all of the analyzed parameters.

The efficacy of the features was further ascertained through machine learning methodologies, thus validating the relevance of the majority of the features that had been designed. The optimal model, a Random Forest classifier, demonstrated the capacity to accurately identify Asn residue reactivity (having an AUC value of 0.87 and an MCC value of 0.84) and to differentiate between AsnGly hotspots, relying on the majority of the features. Among these, conformational parameters, solvation, and the environment electrostatics emerged as key contributors. The findings of this work suggest that the set of structural dynamics features employed effectively captures the different reactivities of Asn residues in six proteins, demonstrating a potential generalizability of the method. Even though the computational effort is not negligible, it is considerably reduced compared to a comprehensive characterization of the reaction rates. Consequently, this framework may be integrated into stable tools to facilitate the semiquantitative prediction of Asn residue reactivity.

## Supplementary Material



## Data Availability

All the necessary files to reproduce our data are openly available at https://zenodo.org/records/16948656?preview=1&token=eyJhbGciOiJIUzUxMiJ9.eyJpZCI6IjQwMjA5ZjZhLTQ3YTctNDU3My1hNzAyLWM2MjVkOWFjNDk1OCIsImRhdGEiOnt9LCJyYW5kb20iOiIyZWIyYzVlYTY4OTcxMzY3NWFmZmExZmQ3ZDRlZmQ4NCJ9.cqaPesZ-xjkHX-TDfWnelyfs4B0Tv7a10JmJ5rjsSk_EDH0hGmpYaDjopDh3EspcWNUzlWuOeX6h8jHZkRWpMQ.

## Notes

Details on the deamidation-prone Asn residues within the investigated proteins, raw data for the ${Vol}_{\mathrm{E⃗}}$ descriptor calculated on B2M Asn residues, B2M-Asn17Gly and B2M-Asn42Gly KDE distributions of the set of parameters commonly employed in analyzing conformational activation, convergence of B2M local PCA among replicates, free energy profiles for B2M Asn-*n* + 1 fragments along PC1 using either R or R* as the reference structure, per-run conformational free energy profiles for B2M, distribution of normalized features across the data set, reactivity-like behavior of deamidating Asn residues within the data set, performance metrics for the RF, LG, and NB models on three additional data set splits, along with average values, raw data for the deamidation score, leave-one-feature-out (LOFO) analysis for each descriptor, and Pearson correlation matrix of features (PDF)
