# Supplementary material for: Mechanism-Driven Features Enable Asn Deamidation Reactivity Prediction via Machine Learning Methods
Source: J Chem Inf Model. 2025 Sep 19;65(19):10588–99. doi: 10.1021/acs.jcim.5c01386 (PMC12529760; doi:10.1021/acs.jcim.5c01386)
Supplement: Supplementary file 1 [file ci5c01386_si_001.pdf]

Supporting Information for: Mechanism-Driven Features Enable Asn Deamidation Reactivity Prediction via Machine Learning Methods

Maria Laura De Sciscio,[†] Rosa De Troia,[†] Joann Kervadec,[‡] Fabio Centola,[¶] Simona Saporiti,[¶] Muriel Priault,[§] and Marco D'Abramo[*,†]

†Department of Chemistry, University of Rome, Sapienza, P.le A. Moro 5, 00185 Rome, Italy

‡CNRS, Université de Bordeaux, UMR 5095, Institut de Biochimie et de Génétique Cellulaires, Bordeaux, France.

¶Analytical Excellence and Program Management, Merck Serono S.p.A., Rome, Italy

§ CNRS, Université de Bordeaux, UMR 5095, Institut de Biochimie et de Génétique Cellulaires, Bordeaux, France.

E-mail: marco.dabramo@uniroma1.it

Table S1: Summary of Asn residues investigated in this work. The PDB code utilized as the starting structure in the MD simulations, along with the Asn reactive residues, are reported.* Asn deamidating under physiological conditions.

Protein	PDB code	nr. ASN	nr. deamidated Asn*	n+1	Refs
B2M	2d4f[1]	5	1 (Asn17)	Gly	[2,3]
GH	1hgu[4]	9	2 (Asn149, Asn152)	Ser, Asp	[5,6]
RNAse	1fs3[7]	10	1 (Asn67)	Gly	[8–10]
SPA	1dee[11]	7	2 (Asn23, Asn28)	Glu, Gly	[12–14]
TPI (dimer)	1r2r[15]	18	4 (Asn15A, Asn71A, Asn15B, Asn71B)	Gly, Gly	[16,17]
trypsin	3aav[18]	15	3 (Asn31, Asn77, Asn97)	Ser, Ser, Ser	[19]

Table S2: Volume of the three-dimensional space occupied by the electric field generated by protein and solvent around Asn-n+1 backbone segment, utilized as a quantitative descriptor of the local electrostatic perturbation on the N-H amide bond. These values are computed using the three replicates. The convergence of the electric field spread among the different runs is assessed by comparing the number of occupied bins within the 50x50x50 grid (see Methods for further details).

N-H amide	Volume	Occupied Bins Run1	Occupied Bins Run2	Occupied Bins Run3
Asn17Gly	1.2123	11748	7543	10295
Asn21Phe	0.9951	8926	8731	9463
Asn24Cys	0.7920	7836	6430	7693
Asn42Gly	0.6956	7464	6921	7170
Asn83His	1.0657	9882	9195	9469

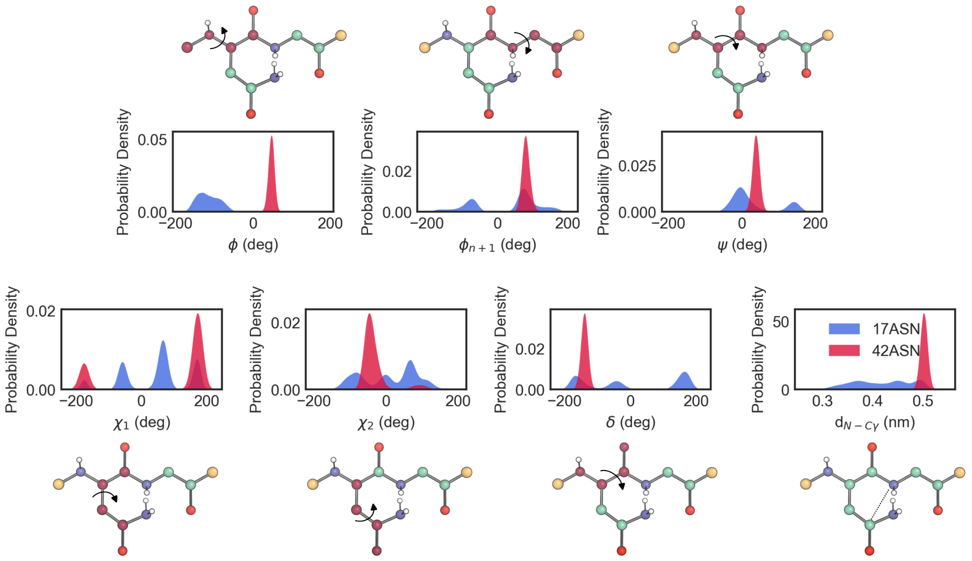

Figure S1: KDE of conformational descriptors adopted to capture deamidation-like behavior in B2M canonical motifs (AsnGly). For each parameter, the atoms forming the dihedral are colored purple in the AsnGly structure reported at the top or the bottom of each graph, while the arrow indicates the bond rotation. The distance is represented as a black dashed line.

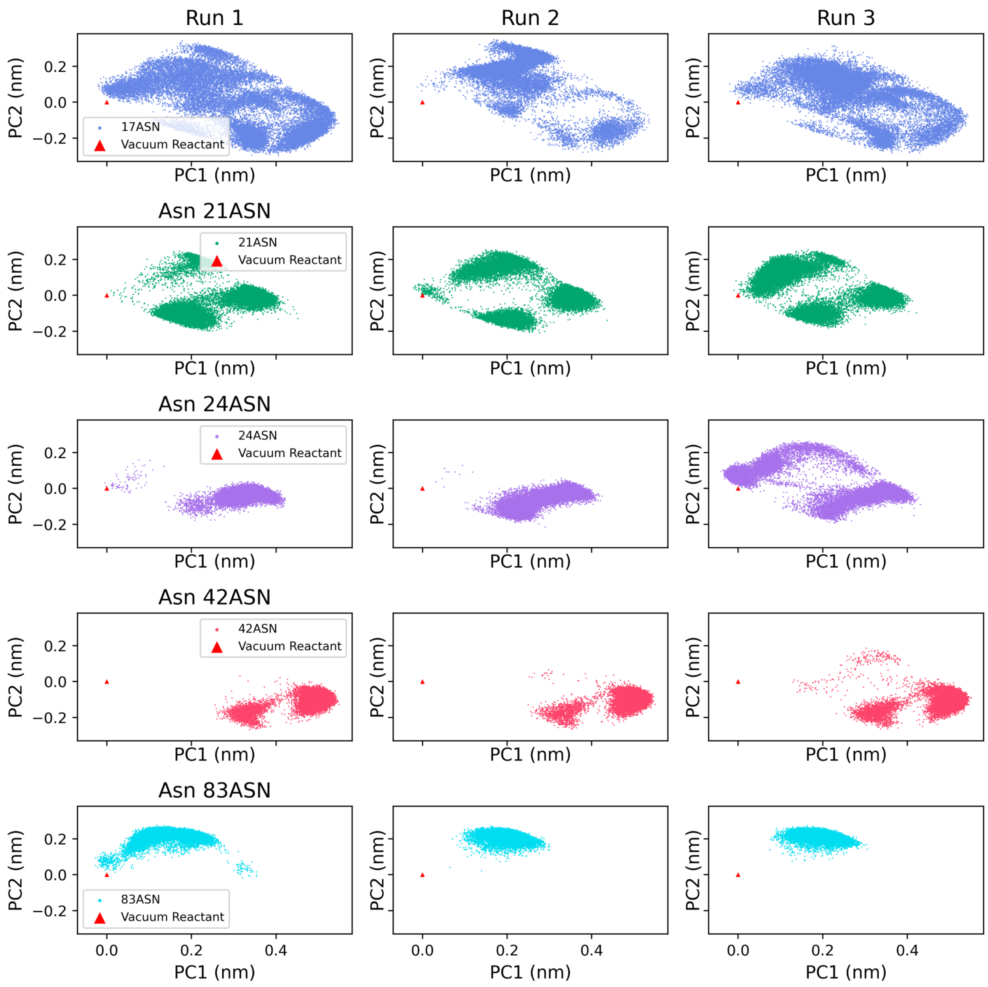

Figure S2: Projection of per-run B2M Asn-n+1 sampled conformation into the first two principal components (PC1 and PC2), representing more than 75% of the total variance of the system, built on AsnGly reactive conformation (R).

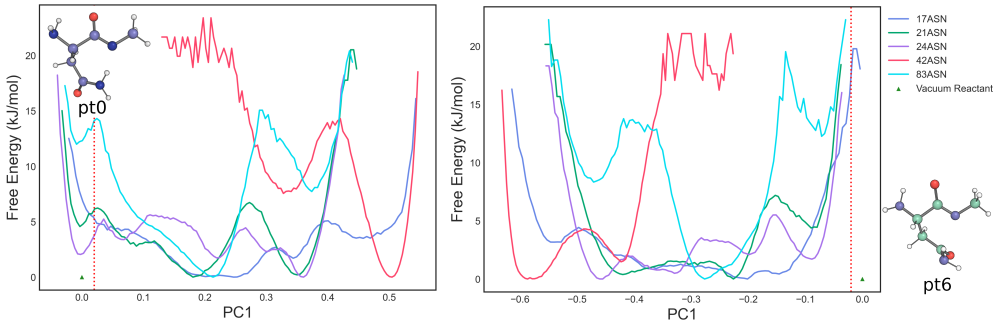

Figure S3: Free energy profile along the first principal component (PC1), defining the conformational subspace built with respect to the QM reactant state (left, R) and the sixth point along the reaction coordinate of the ring-closure step (right, R*). In each plot, the structure of the vacuum reactants (red triangle marker) is illustrated.

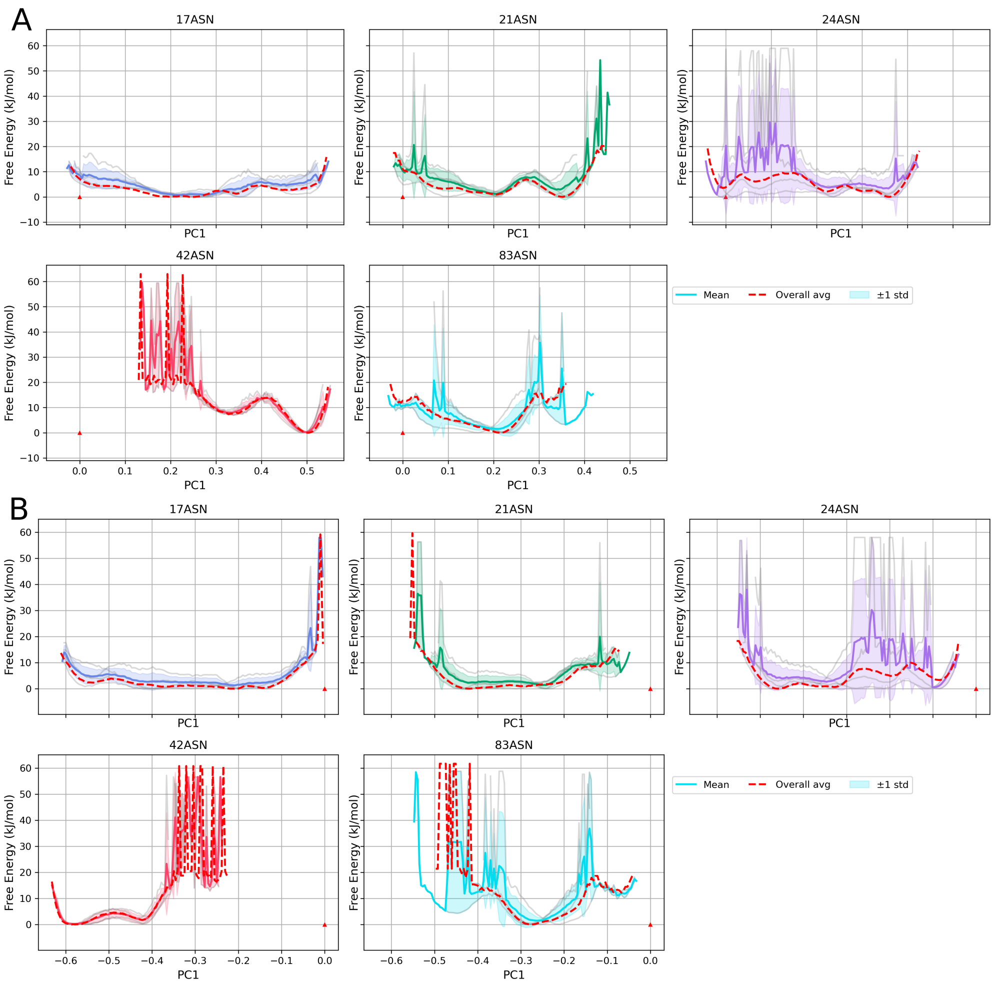

Figure S4: Per-run free energy estimation for B2M along the first principal component (PC1), defining the conformational subspace with respect to R state (A) and R* state (B), illustrated as a red triangle. For each Asn residue, in each subplot, the free energy obtained for each independent run (gray line), the average free energy profile (solid line), the standard deviation (shown as a shaded area), and the overall free energy profile computed on the concatenated trajectories (dotted red line) are reported.

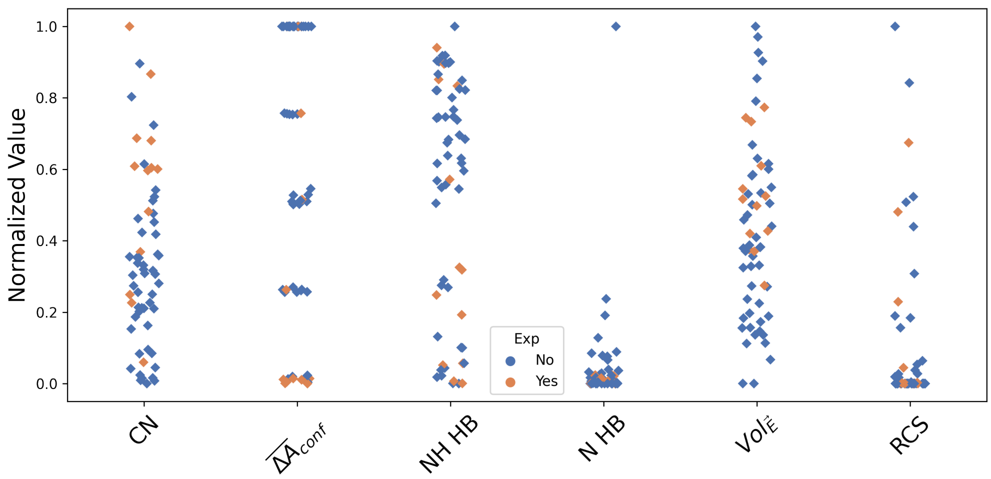

Figure S5: Distribution of normalized feature values for reactive (Exp = Yes, orange) and non-reactive (Exp = No, blue) residues across the entire dataset.

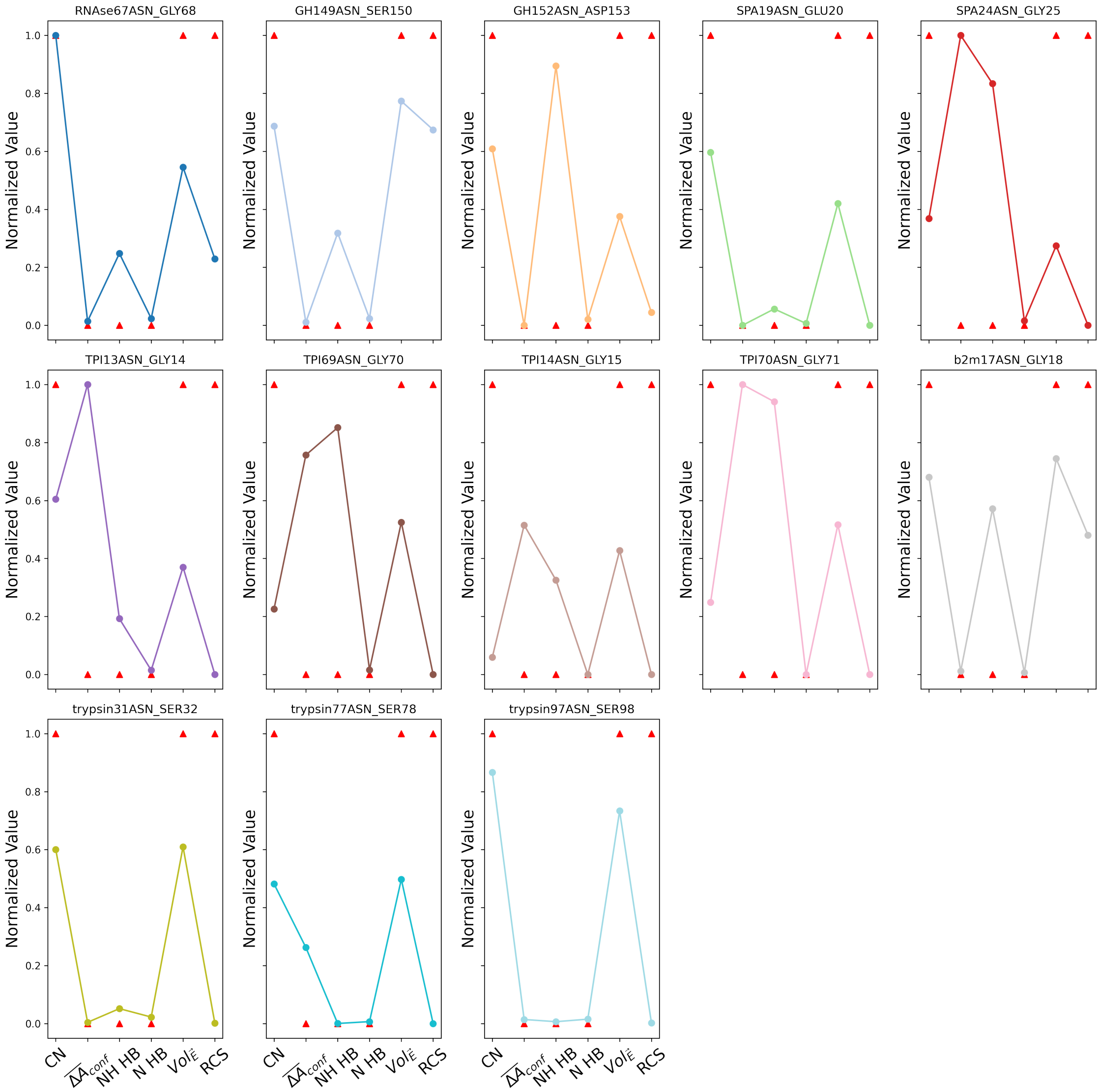

Figure S6: Reactivity-like behavior of deamidating Asn residues within the dataset. For each feature, the chemically-based ideal value is illustrated as a red (triangle) marker in the normalized feature space, as obtained by applying the MinMaxScaler. A high value of CN is expected to promote the deprotonation reaction by facilitating the diffusion of the deprotonating base to the amide NH group and by potentially stabilizing the conjugate base. A high hydrogen-bond frequency could reflect the presence of structural constraints, potentially weakened by the deprotonation reaction. The electric field spread ($Vol_{\vec{E}}$) encodes environmental electrostatic fluctuations potentially enhancing acidity; conformational free energy ($\overline{\Delta A}_{conf}$) characterizes the accessibility of reactive conformations (low descriptor value represents fragment conformationally activated), while the RCS describes the environment's energetic contribution to the ring-closure step (higher value are assigned to fragments conformationally activated and with a favorable neighboring environment).

Table S3: Statistics for predictions of RF, LR, and NB on the two additional test sets. For each classifier, the average and standard deviation (std) are calculated on the three trials here reported, plus the best results reported in Table 1. Precision, F1-score, and Recall are obtained from non-weighted averages on both reactive and non-reactive class predictions. MCC = Matthew's Correlation Coefficient (MCC), and AUC = Area Under the Receiving Operating Curve (ROC).

Model	Trial	Precision	Recall	Accuracy	F1-score	AUC	MCC
RF	1	0.97	0.88	0.95	0.91	0.82	0.84
RF	2	0.97	0.88	0.95	0.91	0.85	0.84
RF	Mean ± std	0.97 ± 0.00	0.88 ± 0.00	0.95 ± 0.00	0.91 ± 0.00	0.85 ± 0.02	0.84 ± 0.00
NB	1	0.62	0.68	0.63	0.59	0.88	0.29
NB	2	0.51	0.52	0.53	0.47	0.47	0.03
NB	Mean ± std	0.60 ± 0.07	0.65 ± 0.09	0.63 ± 0.09	0.58 ± 0.09	0.73 ± 0.19	0.24 ± 0.16
LR	1	0.56	0.58	0.63	0.55	0.63	0.14
LR	2	0.57	0.56	0.74	0.56	0.45	0.13
LR	Mean ± std	0.66 ± 0.14	0.66 ± 0.13	0.73 ± 0.09	0.63 ± 0.11	0.64 ± 0.15	0.28 ± 0.20

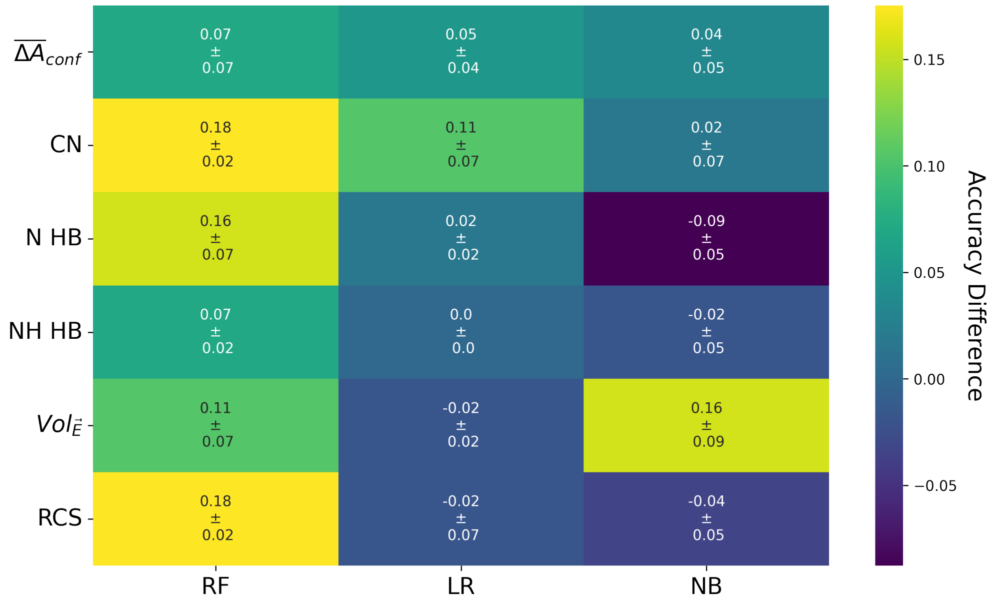

Figure S7: Average LOFO drop in accuracy for the three classifiers employed in the work across the three dataset splits. For feature explanation, please refer to Table 1 and the Methods section.

Table S4: Deamidation score obtained for the best test set as prediction probability using NB, LR, and RF models. The experimental binary result ('Exp') is assigned according to the literature data, reported in Table S1.

Exp	protein	Res	scoreNB	scoreLR	scoreRF	n+1
0	GH	99ASN	0.0000	0.7495	0.3980	SER100
1	trypsin	31ASN	0.9973	0.9118	0.8975	SER32
0	TPI	19ASN	0.1910	0.2472	0.2152	LEU20
1	b2m	17ASN	0.9865	0.7003	0.5870	GLY18
0	TPI	63ASN	0.7806	0.3932	0.2950	CYS64
0	trypsin	159ASN	0.0646	0.1251	0.1724	MET160
0	trypsin	61ASN	0.0000	0.3593	0.3835	GLU62
0	TPI	9ASN	0.0000	0.0359	0.0287	TRP10
1	TPI	70ASN	0.1421	0.2884	0.2167	GLY71
0	trypsin	79ASN	0.5155	0.0766	0.4533	THR80
0	TPI	244ASN	0.0445	0.1021	0.1238	ALA245
0	trypsin	211ASN	0.0217	0.2639	0.2274	TYR212
1	GH	152ASN	0.9609	0.5553	0.5043	ASP153
0	SPA	17ASN	0.3588	0.2375	0.2117	LEU18
0	trypsin	19ASN	0.0000	0.1502	0.1456	SER20
0	GH	12ASN	0.0043	0.0609	0.0912	ALA13
0	b2m	42ASN	0.9964	0.9082	0.4762	GLY43
0	TPI	151ASN	0.7041	0.2948	0.2424	VAL152
0	RNAse	103ASN	0.4490	0.2915	0.4124	LYS104

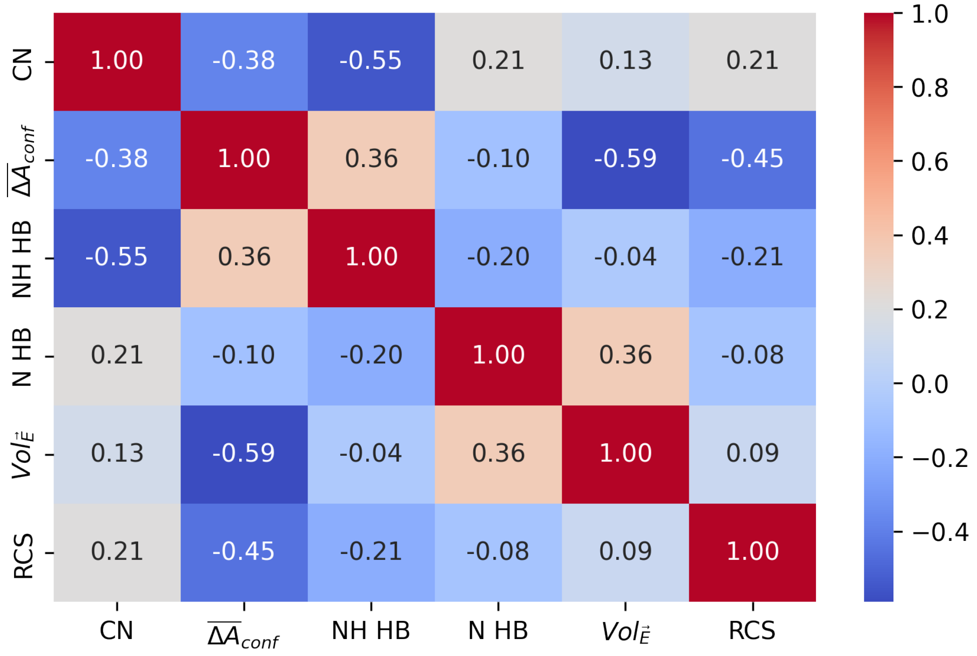

Figure S8: Pearson correlation matrix of the mechanism-based features.

References

(1) Kihara, M.; Chatani, E.; Iwata, K.; Yamamoto, K.; Matsuura, T.; Nakagawa, A.; Naiki, H.; Goto, Y. Conformation of Amyloid Fibrils of β2-Microglobulin Probed by Tryptophan Mutagenesis. *J. Biol. Chem.* **2006**, *281*, 31061–31069.

(2) Fukuda, M.; Takao, T. Quantitative Analysis of Deamidation and Isomerization in beta-Microglobulin by ^{18} O Labeling. *Anal. Chem.* **2012**, *84*, 10388–10394.

(3) Soulby, A. J.; Heal, J. W.; Barrow, M. P.; Roemer, R. A.; O'Connor, P. B. Does deamidation cause protein unfolding? A top-down tandem mass spectrometry study. *Protein Sci* **2015**, *24*, 850–860.

(4) Chantalat, L.; Jones, N.; Korber, F.; Navaza, J.; Pavlovsky, A. The crystal structure of wild-type growth hormone at 2.5 A resolution. *Protein Pept. Lett.* **1995**, *2*, 333–340.

(5) Lewis, U.; Singh, R.; Bonewald, L.; Seavey, B. Altered proteolytic cleavage of human growth hormone as a result of deamidation. *J. Biol. Chem.* **1981**, *256*, 11645–11650.

(6) Karlsson, G.; Eriksson, K.; Persson, A.; Månsson, H.; Söderholm, S. The Separation of Recombinant Human Growth Hormone Variants by UHPLC. *J. Chromatogr. Sci.* **2013**, *51*, 943–949.

(7) Chatani, E.; Hayashi, R.; Moriyama, H.; Ueki, T. Conformational strictness required for maximum activity and stability of bovine pancreatic ribonuclease A as revealed by crystallographic study of three Phe120 mutants at 1.4 Å resolution. *Prot. Sci.* **2002**, *11*, 72–81.

(8) Fagagnini, A.; Montioli, R.; Caloiu, A.; Ribó, M.; Laurents, D. V.; Gotte, G. Extensive deamidation of RNase A inhibits its oligomerization through 3D domain swapping. *Biochim. Biophys. Acta - Proteins Proteom.* **2017**, *1865*, 76–87.

(9) Esposito, L.; Vitagliano, L.; Sica, F.; Sorrentino, G.; Zagari, A.; Mazzarella, L. The ultrahigh resolution crystal structure of ribonuclease A containing an isoaspartyl residue: hydration and sterochemical analysis1. *J. Mol. Biol.* **2000**, *297*, 713–732.

(10) Zabrouskov, V.; Han, X.; Welker, E.; Zhai, H.; Lin, C.; van Wijk, K. J.; Scheraga, H. A.; McLafferty, F. W. Stepwise Deamidation of Ribonuclease A at Five Sites Determined by Top Down Mass Spectrometry. *Biochem.* **2006**, *45*, 987–992.

(11) Graille, M.; Stura, E. A.; Corper, A. L.; Sutton, B. J.; Taussig, M. J.; Charbonnier, J.-B.; Silverman, G. J. Crystal structure of a Staphylococcus aureus protein A domain complexed with the Fab fragment of a human IgM antibody: Structural basis for recognition of B-cell receptors and superantigen activity. *PNAS* **2000**, *97*, 5399–5404.

(12) Kanje, S.; Scheffel, J.; Nilvebrant, J.; Hober, S. In *Approaches to the Purification, Analysis and Characterization of Antibody-Based Therapeutics*; Matte, A., Ed.; Elsevier, 2020; pp 35–54.

(13) Xia, H.-F.; Liang, Z.-D.; Wang, S.-L.; Wu, P.-Q.; Jin, X.-H. Molecular Modification of Protein A to Improve the Elution pH and Alkali Resistance in Affinity Chromatography. *Appl Biochem Biotechnol* **2014**, *172*, 4002–4012.

(14) Linhult, M.; Gülich, S.; Gräslund, T.; Simon, A.; Karlsson, M.; Sjöberg, A.; Nord, K.; Hober, S. Improving the tolerance of a protein a analogue to repeated alkaline exposures using a bypass mutagenesis approach. *Proteins:Struct., Funct., Bioinf.* **2004**, *55*, 407–416.

(15) Aparicio, R.; Ferreira, S. T.; Polikarpov, I. Closed Conformation of the Active Site Loop of Rabbit Muscle Triosephosphate Isomerase in the Absence of Substrate: Evidence of Conformational Heterogeneity. *J. Mol. Biol.* **2003**, *334*, 1023–1041.

(16) Ugur, I.; Marion, A.; Aviyente, V.; Monard, G. Why Does Asn71 Deamidate Faster Than Asn15 in the Enzyme Triosephosphate Isomerase? Answers from Microsecond

Molecular Dynamics Simulation and QM/MM Free Energy Calculations. *Biochem.* **2015**, *54*, 1429–1439.

(17) Yüksel, K.; Gracy, R. W. In vitro deamidation of human triosephosphate isomerase. *Archives of Biochem and Biophysics* **1986**, *248*, 452–459.

(18) Iyaguchi, D.; Kawano, S.; Takada, K.; Toyota, E. Structural basis for the design of novel Schiff base metal chelate inhibitors of trypsin. *BMCL* **2010**, *18*, 2076–2080.

(19) Kossiakoff, A. A. Tertiary Structure is a Principal Determinant to Protein Deamidation. *Science* **1988**, *240*, 191–194.
